# Calycosin suppresses the activating effect of granulocyte-macrophage-colony-stimulating factor-producing T helper cells on macrophages in experimental atherosclerosis

**DOI:** 10.3389/fphar.2025.1607349

**Published:** 2025-07-10

**Authors:** Xiaofang Xiong, Wei Huang, Xuexue Yang, Xun Wang, Beibei Wu, Dongsheng Li

**Affiliations:** ^1^ The Department of Cardiology at Wuhan Third Hospital, Wuhan, Hubei, China; ^2^ The Department of Traditional Chinese Medicine at Wuhan Third Hospital, Wuhan, Hubei, China

**Keywords:** calycosin, granulocyte-macrophage-colony-stimulating factor-producing T helper cells, atherosclerosis, macrophages, nuclear receptor subfamily 4 group A member 3

## Abstract

**Background:**

T cells are contributors to atherosclerosis pathogenesis. Granulocyte-macrophage-colony-stimulating factor (GM-CSF)-producing T helper (ThGM) cells, a specialized helper T cell subset that highly expresses GM-CSF but lacks other helper T cell markers, could exacerbate atherosclerosis development. Calycosin has been reported to suppress atherosclerosis progression. However, the effect of calycosin on ThGM cells is unknown. This study was designed to test the calycosin-induced impact on the pro-atherosclerotic function of ThGM cells in a mouse atherosclerosis model.

**Methods:**

Apolipoprotein E knockout (ApoE^−/−^) mice were fed a high-fat diet and calycosin. The phenotype and cytokine expression of aortic ThGM cells were assessed by flow cytometry. Calycosin-derived influences on ThGM cell differentiation, proliferation, and function were determined by flow cytometry, quantitative RT-PCR, Immunoblotting, gene silencing assays, and co-culture with macrophages.

**Results:**

Aortic ThGM cell frequency was attenuated after calycosin administration. Live aortic ThGM cells, phenotypically featuring CD4^+^CCR6^−^CCR8^−^CXCR3^−^CCR10^+^, showed slower proliferation and weaker macrophage-activating capability in calycosin-treated mice. Besides, calycosin repressed *in vitro* ThGM cell differentiation and subsequently impaired ThGM cell-mediated macrophage activation, oxidized low-density lipoprotein (Ox-LDL) uptake, and foam cell formation. Importantly, calycosin upregulated nuclear receptor subfamily 4 group A member 3 (NR4A3) in ThGM cells. NR4A3 silencing partially restored the function of calycosin-treated ThGM cells.

**Conclusion:**

Calycosin inhibits ThGM cell activity to suppress ThGM-cell-mediated activation of pro-atherosclerotic macrophages to ultimately ameliorate atherosclerosis progression. Therefore, we revealed a novel mechanism by which calycosin protects against atherosclerosis.

## 1 Introduction

Inflammation plays an important role in atherosclerosis development (Markin et al., 2020). In particular, T lymphocytes are active pro-inflammatory cells that contribute to atherosclerosis progression and destabilize plaque (Wolf and Ley, 2019). Under the instructions of innate immune cells such as dendritic cells and macrophages, CD4^+^ T cells become effector T cells such as T helper 1 (Th1) and T helper 17 (Th17) cells to exert pro-inflammatory effects. By initiating, maintaining, and promoting chronic inflammation in atherosclerotic areas, Th1 or Th17 cells exacerbate foam cell generation and plaque formation (Gao et al., 2010; Taleb et al., 2015; Brauner et al., 2018; Ruiz de Morales et al., 2020; Wang et al., 2021; Chaouhan et al., 2022).

In the past decade, a novel CD4^+^ T cell subpopulation was found to release a large quantity of granulocyte macrophage-colony-stimulating factor (GM-CSF) with few IFN-γ or IL-17 in autoimmune disorders (Zhang et al., 2013; Noster et al., 2014). Named GM-CSF-producing T helper (ThGM) cells, these T cells are devoid of T-box expressed in T cells (T-bet) and retinoic acid-related orphan receptor gamma t (RORγt), making them unique to other known helper T cell subsets. Besides, ThGM cells produce interleukin-2 (IL-2), interleukin-3 (IL-3), tumor necrosis factor (TNF), and CC chemokine ligand 20 (CCL20) to support the activities of other T helper cells (Zhang et al., 2013; Rasouli et al., 2020). Interleukin-7 (IL-7) has been discovered to induce ThGM cell generation and pathogenicity (Sheng et al., 2014). Recent research implies that ThGM cells can polarize towards Th1 cells under the influence of IL-12 (Noster et al., 2014; Rasouli et al., 2020). A set of chemokine receptors, such as CCR10, CCR4, CXCR3, CCR6, and CCR8, can be used to distinguish ThGM cells from Th1, Th2, and Th17 cells (Noster et al., 2014; Stadhouders et al., 2018; Miyagawa and Asada, 2021). Our previous study characterized the presence and activity of ThGM cells in a mouse atherosclerosis model (Xiong et al., 2024). Importantly, we found that live aortic ThGM cells are phenotypically CCR6^−^CCR8^−^CXCR3^−^CCR10^+^ and can activate macrophages to produce interleukin-1β (IL-1β), TNF, interleukin-6 (IL-6), and C-C motif chemokine ligand 2 (CCL2), suggesting that aortic ThGM cells might be involved in macrophage-mediated atherosclerosis development (Xiong et al., 2024). Nonetheless, the significance and regulatory mechanism of atherosclerotic ThGM cells remain elusive.

As an O-methylated isoflavone produced in Trifolium pratense L, calycosin has the potential to treat malignant cancers, inflammatory diseases, ischemia, and cardiovascular disorders (Gao et al., 2014). Calycosin has been revealed to attenuate oxidative stress by decreasing malondialdehyde (MDA), protein carbonyl, and reactive oxygen species (ROS) while promoting the function of glutathione peroxidase (GSH-Px) and superoxide dismutase (SOD) (Deng et al., 2021; Gong et al., 2021). Interestingly, the anti-atherosclerosis efficacy of calycosin has been unveiled in recent years. Calycosin enhances the macrophage autophagy to improve high-fat diet-caused atherosclerosis (Ma et al., 2022). Calycosin activates the AMPK/mTOR signaling to ameliorate autophagy stoppage in smooth muscle cells to mitigate vascular calcification (Zhou et al., 2023). Data mining research implies that calycosin might modulate nucleolus transcription factors to suppress inflammatory reactions and oxidative stress in atherosclerosis (Xu et al., 2024). These studies strongly suggest that calycosin could impede plaque formation by multiple mechanisms. However, the effect of calycosin on atherosclerotic T cells has not been uncovered.

The nuclear receptor subfamily 4 group A family contains three orphan nuclear receptors NR4A1, NR4A2 and NR4A3. They function as transcription factors and their activity is regulated via their expression levels rather than ligand binding (Odagiu et al., 2020b). NR4A proteins contribute to T cell anergy or exhaustion since the artificial overexpression of each NR4A protein leads to an exhaustion-like transcriptome profile in T cells (Yoshimura et al., 2021). Moreover, NR4A3 seems to be a crucial regulator of CD8^+^ T cell responses, because NR4A3 deficiency results in upregulation of cytokine production (Odagiu et al., 2020a). Nonetheless, whether calycosin can alter NR4A protein expression to impact T cell response has not been investigated.

In this study, we evaluated whether calycosin influences the pro-inflammatory activities of both aortic Th1 and ThGM cells. We found that calycosin had a stronger suppressive effect on ThGM cells than Th1 cells. Importantly, calycosin inhibited ThGM cell-induced activation of atherosclerotic macrophages. Therefore, we revealed a novel mechanism by which calycosin protects against atherosclerosis.

## 2 Materials and methods

### 2.1 Atherosclerosis induction and calycosin treatment

The study was approved by the Wuhan Third Hospital Animal Care and Use Committee and implemented abiding by the Wuhan Third Hospital Guidelines for the Use of Animals. Calycosin (Cat# HY-N0519, MedChemExpress) stock solution was prepared in dimethyl sulfoxide (DMSO), and calycosin working solution was prepared in saline containing 1% dimethyl sulfoxide (DMSO). Eight-week-old male apolipoprotein E knockout (ApoE^−/−^) mice (Beijing Biocytogen Co., Ltd) were fed regular chow or a high-fat diet (0.2% cholesterol and 21% fat) with or without a daily oral gavage of calycosin (60 mg/kg body weight) for 16 weeks. No significant signs of distress, weight loss, and changes in activity were observed during the entire induction period. The control mice received an equal volume of normal saline. To substantiate atherosclerotic lesion formation, the mice were euthanized with CO_2_ to harvest the aortas. The aortas were fixed in 4% formaldehyde, incubated in 5% oil red O (Abcam) for half an hour, and washed with running water before observation on a microscope.

### 2.2 Enriching aortic leukocytes

All relevant reagents were purchased from Sigma-Aldrich. Aortic leukocytes were collected using previously established protocols (Matthew et al., 2011; Breanne et al., 2015). Immediately after euthanization with CO_2_, each animal was perfused with 3 mL of phosphate-buffered saline (PBS) through the cardiac puncture, followed by harvesting the aorta and associated branches. The harvested tissues were cut into small pieces and then immersed in 0.2 mL of RPMI1640 supplemented with 10% fetal calf serum (FCS), 50 U/ml DNase I, 150 U/ml collagenase XI, 400 U/ml collagenase I, and 50 U/ml hyaluronidase I-s for 30 min with frequent agitation at 37°C. The tissues were then gently pressed through a 40-μm cell strainer to prepare single-cell suspensions. The cells were washed and resuspended in 0.2 mL of PBS before further processing. To acquire sufficient cells in some experiments, the aortas of three–five animals were pooled for leukocyte enrichment.

For intracellular cytokine detection in T cells, enriched leukocytes or T cells were stimulated with 10 ng/mL phorbol 12-myristate 13-acetate (PMA) and 500 ng/mL ionomycin along with 10 μg/mL brefeldin A for 4 hours, followed by surface antigen staining and intracellular protein staining procedures.

### 2.3 Flow cytometry

Flow cytometry antibody information is provided in Supplementary Table S1. Cell surface and cytosolic proteins were detected as described in our previous research (Xiong et al., 2024). Cells were analyzed on a BD LSRII flow cytometer or sorted on a BD FACSAria cell sorter (BD Biosciences).

### 2.4 Quantitative reverse transcription and polymerase chain reaction (q-RT-PCR)

The Animal Tissue/Cell RNA Purification Kit (Cat# G3640-50T, Servicebio) was used to extract RNAs from macrophages. The SweScript RT II First Strand cDNA Synthesis Kit (Cat# G3333-50, Servicebio) was used to synthesize cDNAs. Quantitative PCR was executed using the 2×Universal Blue SYBR Green qPCR Master Mix (Cat# G3328-01, Servicebio) on a LightCycler^®^ 480 System (Roche). The primers are displayed in Supplementary Table S2. The fold changes of the mRNAs of interest were computed using the 2^−ΔΔCt^ method (Livak and Schmittgen, 2001).

### 2.5 Enriching macrophages from the spleen

Macrophages were sorted from mouse spleens using the EasySep™ Mouse F4/80 Positive Selection Kit (Cat# 100–0659, STEMCELL Technologies) based on the manufacturer’s brochure.

### 2.6 *In vitro* ThGM cell generation

CD4^+^CD25^−^ T cells were enriched from the spleens of wild-type C57BL/6J mice using the Mouse Naïve T Cell Isolation Kit (Cat# ab322030, Abcam) following the supplier’s instructions. ThGM cell induction was carried out following previous studies (Sheng et al., 2014; Lu et al., 2018; Rasouli et al., 2022). Briefly, 1.0 × 10^6^/mL CD4^+^CD25^−^ T cells were cultured in RPMI 1640 supplemented with 10% FCS in the presence of 5 μg/mL pre-coated CD3ε antibody (145-2C11, eBioscience), 2 μg/mL soluble CD28 antibody (37.51, eBioscience), 10 μg/mL IFN-γ neutralizing antibody (MAB485-SP, R&D Systems), 10 μg/mL IL-12 neutralizing antibody (AF-419-NA, R&D Systems), 5 μg/mL IL-4 neutralizing antibody (AF-404-SP, R&D Systems), and 2 ng/mL recombinant murine IL-7 (407-ML-025/CF, R&D Systems) for 4 days. To evaluate the effects of calycosin on ThGM cell differentiation, calycosin was added at the start of the induction at the indicated concentrations. During the last 4 hours of the induction, 50 ng/mL PMA, 1 μg/mL ionomycin, and 10 μg/mL Brefeldin A were added to the culture. After that, cells were subjected to intracellular cytokine staining to detect GM-CSF and IFN-γ expression. To appraise T cell proliferation, CD4^+^CD25^−^ T cells were labeled with 5 μM carboxyfluorescein succinimidyl ester (CFSE, Cat# 423801, BioLegend) before the induction.

### 2.7 T cell-macrophage co-culture

Equal volumes of 2 × 10^4^/ml flow cytometry-sorted aortic T cells and 2 × 10^4^/ml macrophages were mixed in RPMI1640 supplemented with 10% FCS. The mixture was seeded into a 96-well v-bottom microplate (Corning) to incubate for 24 h with 5 μg/mL pre-coated CD3ε antibody and 2 μg/mL soluble CD28 antibody. After that, the microplate was vigorously shaken and floating cells (i.e., T cells) were carefully removed. Adherent cells (mostly macrophages) were used for q-RT-PCR.

Regarding *in vitro* induced ThGM cells, equal volumes of 3 × 10^6^/ml ThGM cells and 1 × 10^6^/ml splenic macrophages were mixed and cultured in a 96-well round-bottom microplate (Corning) for 24 h with the same agonistic antibodies as above. In some experiments, recombinant mouse GM-CSF (Cat# 415-ML-005/CF, R&D Systems) was added at the start of the co-culture at a final concentration of 20 ng/mL. At the last 4 hours of the co-culture, 10 μg/mL brefeldin A was added to the culture. Floating T cells were removed the same way as above. Adherent macrophages were lifted after 5-min incubation in 0.25% Trypsin-EDTA solution and were then used for intracellular cytokine detection, oxLDL uptake assay, or foam cell staining.

### 2.8 oxLDL uptake

5 × 10^5^/mL macrophages were incubated in basic RPMI 1640 in the presence of oxLDL-DyLight 488 (1:50 diluted, Cat# 601180, Cayman Chemical) for 120 min at 37°C. After 5-min trypsinization with 0.25% Trypsin-EDTA, macrophages were re-suspended in 0.5 mL of PBS, followed by the addition of 0.5 mL of 0.4% trypan blue (Cat# PB180423, Procell) to extinguish cell surface-bound oxLDL-DyLight 488. The intensity of intracellular oxLDL-DyLight 488 was quantified by flow cytometry.

### 2.9 Quantification of foam cell generation and cholesterol content

Macrophage density was adjusted to 1 × 10^6^/mL, followed by 24-h incubation with 50 μg/mL oxLDL. The subsequent fixation, oil red O staining, and quantification of optical density (OD) of oil red O were implemented as described in our previous study (Xiong et al., 2024). The cholesterol content was evaluated using the Cholesterol Quantification Kit (Cat# E1015, Applygen) according to the manufacturer’s manual.

### 2.10 Immunofluorescent microscopy

After being placed on poly-L-lysine-coated coverslips and air-dried, T cells were subjected to 15-min fixation with 10% paraformaldehyde, 30-min permeabilization with 0.25% Triton X-PBS, and overnight incubation with the NR4A3 antibody (1:100, Cat# ABE1456, Millipore-Sigma). After three washes with PBS, the cells were labeled with 4 μg/mL Alexa Fluo 488-conjugated goat anti-rabbit antibody (Cat# A-11008, ThermoFisher) for 60 min. The fluorescence was observed on a Zeiss AXIO Observer.Z1 inverted microscope.

### 2.11 Immunoblotting

Cellular proteins were extracted by 30-min incubation in ice-cold RIPA buffer (Cat# P0013B, Beyotime) followed by centrifugation at 10,000 *g*. Forty micrograms of proteins from each lysate were used for electrophoresis on a 12% SDS-PAGE gel. The NR4A3 antibody (1:1000, Cat# ABE1456, Millipore-Sigma) was used to determine NR4A3 expression.

### 2.12 Lentiviral transduction

The Nr4a3-set siRNA/shRNA/RNAi Lentivector (Cat# 32143094) and scrambled control siRNA Lentivector (Cat# LV015-G) were purchased from Applied Biological Materials. These lentivectors also carry a GFP-encoding sequence so transduced cells express GFP. The preparation and quantification of lentiviral particles were attained by Viraltherapy Technologies. One day after the *in vitro* ThGM generation, 8 μg/mL polybrene (Millipore-Sigma) and lentiviral particles (multiplicity of infection = 10) were added to incubate T cells overnight. The next morning the medium was replaced with fresh medium containing the same ThGM-inducing reagents to further culture T cells for 3 days. After that, the expression of GFP and NR4A3 was assessed by flow cytometry or q-RT-PCR, respectively. Before detecting intracellular cytokines, T cells were treated with 20 ng/mL PMA, 1 μg/mL ionomycin, and 10 μg/mL brefeldin A at the last 6 h of ThGM induction.

### 2.13 Statistics

All data were shown as mean ± standard deviation. Each experiment was independently conducted two or three times. Student’s t-test or One-Way ANOVA was used to determine statistical significance. A P-value <0.05 was considered significant.

## 3 Results

### 3.1 Calycosin reduces aortic ThGM cell accumulation in atherosclerosis

**FIGURE 1:**
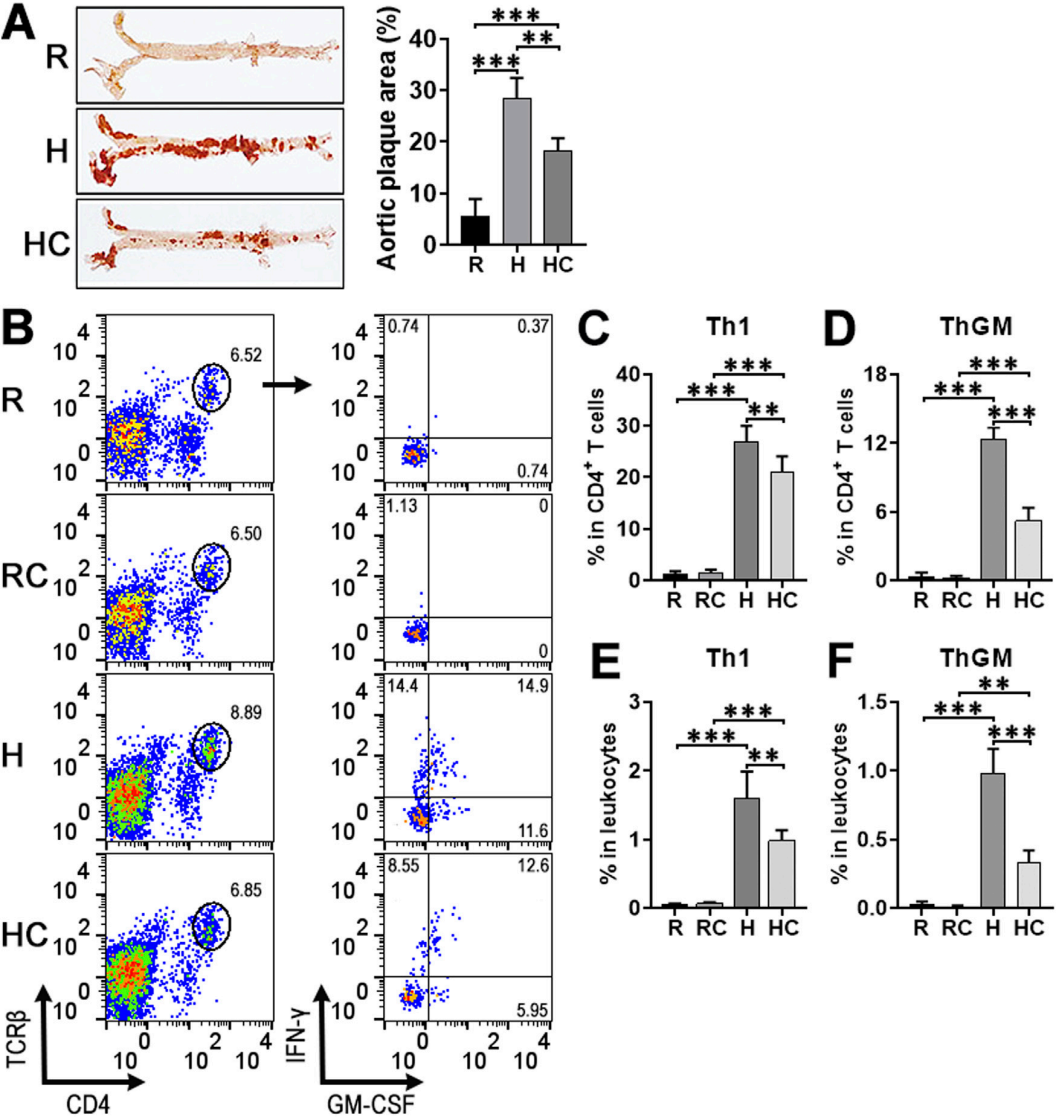
The effect of calycosin on the accumulation of aortic Th1 and ThGM cells in atherosclerosis. **(A)** Atherosclerosis induction at week sixteen after the high-fat diet feeding. Representative Oil red O staining images are shown in the left panel. Aortic plaque area quantification is shown in the right panel. R: ApoE^−/−^ mice fed a regular diet. H: ApoE^−/−^ mice fed the high-fat diet. HC: ApoE^−/−^ mice fed the high-fat diet with calycosin treatment. **(B)** Dot plots illustrating the expression of IFN-γ and GM-CSF in aortic CD4^+^ T cells. Isolated aortic leukocytes were stimulated with PMA + ionomycin + brefeldin A for 4 h followed by surface and intracellular staining. R: ApoE^−/−^ mice fed a regular diet. RC: ApoE^−/−^ mice fed the calycosin-containing regular diet. H: ApoE^−/−^ mice fed the high-fat diet. HC: ApoE^−/−^ mice fed the calycosin-containing high-fat diet. **(C–F)** The frequencies of Th1 (IFN-γ^+^GM-CSF^-^ and IFN-γ^+^GM-CSF^+^) and ThGM (IFN-γ^-^GM-CSF^+^) cells in total CD4^+^ T cells or total leukocytes, respectively. N = 5 mice per group. One-Way ANOVA. **: *P* < 0.01. ***: *P* < 0.001.

To explore the effect of calycosin on atherosclerotic ThGM cells, we established a mouse atherosclerosis model with calycosin treatment. Consistent with previous research (Ma et al., 2022), calycosin significantly reduced plaque formation in the aorta (Figure 1A). We enriched leukocytes from the aortas of ApoE^−/−^ mice. Doublets and dead leukocytes were excluded (Supplementary Figure S1) while TCRβ^+^CD4^+^ cells were selected to determine the expression of IFN-γ and GM-CSF. As shown in Figure 1B, IFN-γ and GM-CSF were scarcely expressed in aortic CD4^+^ T cells of ApoE^−/−^ mice fed a regular diet with or without calycosin, suggesting that calycosin did not induce the generation of either Th1 or ThGM cells in normal aortas. In ApoE^−/−^ mice fed the high-fat diet without calycosin, robust expression of IFN-γ and GM-CSF was observed in aortic CD4^+^ T cells. According to our previous research (Xiong et al., 2024), IFN-γ^+^GM-CSF^-^ and IFN-γ^+^GM-CSF^+^ T cells were designated as Th1 cells, whereas IFN-γ^-^GM-CSF^+^ T cells were designated as ThGM cells. Notably, both the frequencies of aortic Th1 and ThGM cells were elevated in ApoE^−/−^ mice fed the high-fat diet without calycosin, while calycosin treatment moderately decreased aortic Th1 cell frequency and considerably lessened aortic ThGM cell frequency (Figures 1C–F).

### 3.2 Calycosin does not alter chemokine receptor expression on the surface of aortic ThGM cells

**FIGURE 2:**
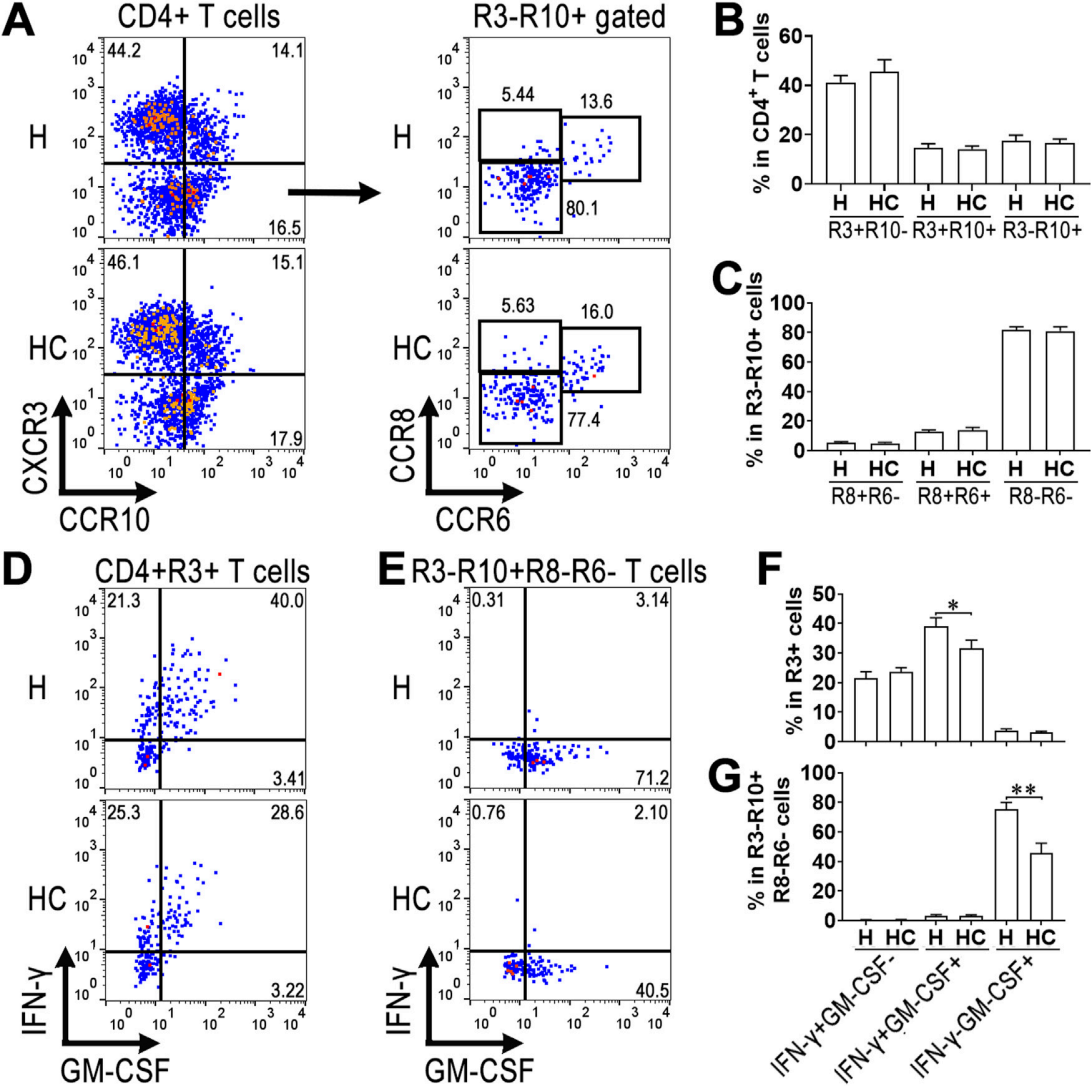
Chemokine receptor expression on aortic ThGM cells. Isolated aortic leukocytes were stimulated with PMA + ionomycin + brefeldin A for 4 h followed by surface and intracellular staining. **(A)** Dot plots showing the expression of CXCR3 and CCR10 on aortic CD4^+^ T cells and the expression of CCR8 and CCR6 on CD4^+^CXCR3^−^CCR10^+^ T cells. H: ApoE^−/−^ mice fed the high-fat diet. HC: ApoE^−/−^ mice fed the calycosin-containing high-fat diet. **(B)** The frequencies of CXCR3^+^CCR10^-^, CXCR3^+^CCR10^+^, and CXCR3^−^CCR10^+^ cells in total aortic CD4^+^ T cells. **(C)** The frequencies of CCR8^+^CCR6^-^, CCR8^+^CCR6^+^, and CCR8^−^CCR6^-^ cells in CD4^+^CXCR3^−^CCR10^+^ T cells. **(D)** Dot plots indicating the expression of IFN-γ and GM-CSF in CD4^+^CXCR3^+^ T cells. **(E)** Dot plots indicating the expression of IFN-γ and GM-CSF in CD4^+^CXCR3^−^CCR10^+^CCR8^−^CCR6^-^ T cells. **(F)** The frequencies of IFN-γ^+^GM-CSF^-^, IFN-γ^+^GM-CSF^+^, and IFN-γ^-^GM-CSF^+^ cells in CD4^+^CXCR3^+^ T cells. **(G)** The frequencies of IFN-γ^+^GM-CSF^-^, IFN-γ^+^GM-CSF^+^, and IFN-γ^-^GM-CSF^+^ cells in CD4^+^CXCR3^−^CCR10^+^CCR8^−^CCR6^-^ T cells. N = 4 samples per group. Each sample contains cells pooled from 4 mice. Student’s t-test. *: *P* < 0.05. **: *P* < 0.01.

We previously identified live aortic ThGM cells as CD4^+^CXCR3^−^CXCR10^+^CCR8^−^CCR6^-^ T cells (Xiong et al., 2024). To determine whether calycosin changes the phenotype of aortic ThGM cells, we evaluated the expression of CXCR3, CXCR10, CCR8, CCR6, IFN-γ, and GM-CSF in aortic CD4^+^ T cells of atherosclerotic mice. As shown in Figures 2A,B, calycosin treatment did not change the frequencies of CXCR3^+^CCR10^-^, CXCR3^+^CCR10^+^, and CXCR3^−^CCR10^+^ cells among total CD4^+^ T cells (Figures 2A,B). Furthermore, the frequencies of CCR8^+^CCR6^-^, CCR8^+^CCR6^+^, and CCR8^−^CCR6^-^ cells among CD4^+^CXCR3^−^CCR10^+^ T cells were not remarkably changed by calycosin treatment (Figures 2A,C). Notably, CD4^+^CXCR3^+^ T cells, including CXCR3^+^CCR10^-^ and CXCR3^+^CCR10^+^ T cells, expressed abundant IFN-γ and GM-CSF, suggesting their Th1 identity (Figures 2D,F). CD4^+^CXCR3^−^CXCR10^+^CCR8^−^CCR6^-^ T cells, however, primarily expressed GM-CSF but not IFN-γ, revealing their ThGM identity (Figures 2E,G). Interestingly, compared with their counterparts in the mice without calycosin treatment, Th1 cells of calycosin-treated mice mildly downregulated the expression of IFN-γ and GM-CSF, whereas ThGM cells of calycosin-treated mice robustly downregulated GM-CSF expression (Figures 2D–G). This implies that calycosin strongly inhibited ThGM cell function while weakly suppressed Th1 function.

### 3.3 Calycosin mitigates the pro-inflammatory effect of aortic ThGM cells on macrophages

**FIGURE 3:**
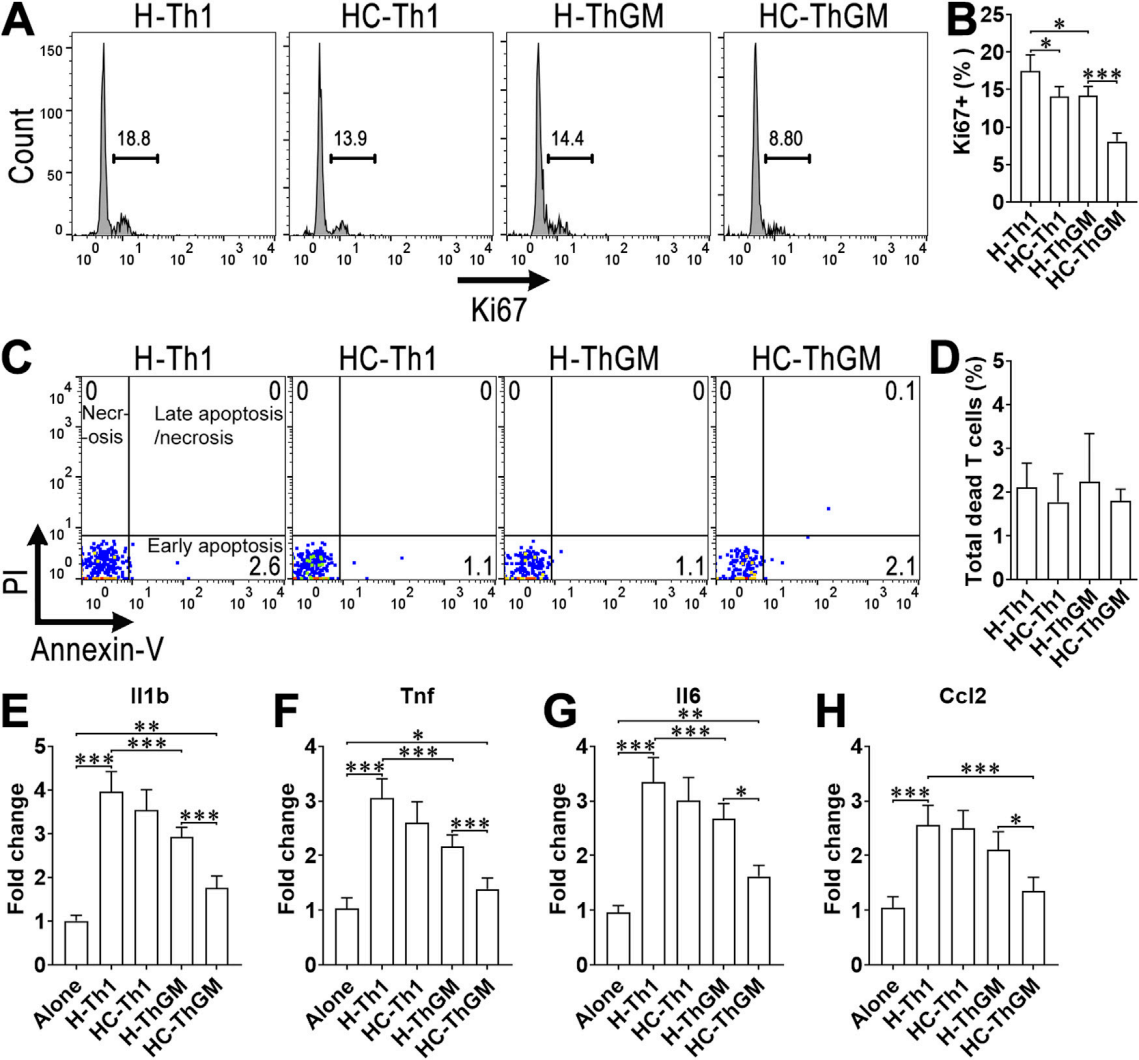
The effect of calycosin on aortic CD4^+^ T cell-induced activation of macrophages. Note that aortic T cell subsets were not stimulated with PMA + ionomycin + brefeldin A in these assays so the results reflect the *in vivo* activities of the T cell subsets. **(A)** Histograms showing Ki67 expression. H-Th1: aortic Th1 cells of mice fed the high-fat diet. HC-Th1: aortic Th1 cells of mice fed the calycosin-containing high-fat diet. H-ThGM: aortic ThGM cells of mice fed the high-fat diet. HC-ThGM: aortic ThGM cells of mice fed the calycosin-containing high-fat diet. **(B)** Ki67^+^ cell frequency in each subset. **(C)** Dot plots showing the apoptosis and necrosis of aortic Th1 and ThGM cells. **(D)** The frequency of total dead T cells (including early apoptotic, late apoptotic, and necrotic cells). **(E–H)** Transcript levels of pro-inflammatory cytokines/chemokines in macrophages after 24-h co-culture with aortic CD4^+^ T cell subsets. Alone: macrophages alone. H-Th1: macrophages co-cultured with Th1 cells of mice fed the high-fat diet. HC-Th1: macrophages co-cultured with Th1 cells of mice fed the calycosin-containing high-fat diet. H-ThGM: macrophages co-cultured with ThGM cells of mice fed the high-fat diet. HC-ThGM: macrophages co-cultured with ThGM cells of mice fed the calycosin-containing high-fat diet. N = 5 samples per group. In **(E–H)**, each sample contains T cells pooled five mice. One-Way ANOVA. *: *P* < 0.05. **: *P* < 0.01. ***: *P* < 0.001.

Calycosin administration mildly lowered Ki67 expression in aortic Th1 cells while profoundly reducing Ki67 expression in aortic ThGM cells, compared with their counterparts in atherosclerotic mice without calycosin treatment (Figures 3A,B). However, the apoptosis and necrosis of aortic Th1 or ThGM cells were not significantly impacted by calycosin (Figures 3C,D). To check the influences of aortic Th1 and ThGM cells on macrophage function, macrophages were enriched from normal mouse spleens and cultured with or without sorted aortic T cells for 24 h at a ratio of 1:1. After that, floating T cells were removed while adherent macrophages were subjected to q-RT-PCR to quantify the transcripts of pro-inflammatory IL-1β, TNF, IL-6, and CCL2 (Supplementary Figure S2). In mice without calycosin treatment, aortic Th1 cells induced substantial upregulation of these cytokines in macrophages. Aortic Th1 cells of calycosin-treated mice induced slightly lower IL-1β, TNF, and IL-6 than their counterparts in mice without calycosin treatment, implying an insignificant impact of calycosin on Th1 cell-mediated macrophage activation (Figures 3E–H). By contrast, in mice without calycosin treatment, aortic ThGM cells moderately boosted cytokine expression in macrophages. Aortic ThGM cells of calycosin-treated mice exhibited a significantly lower macrophage-activating effect than their counterparts in mice without calycosin treatment, as evidenced by less expression of IL-1β, TNF, and IL-6 in macrophages after the co-culture (Figures 3E–H).

### 3.4 Calycosin suppresses ThGM cell generation *in vitro*

**FIGURE 4:**
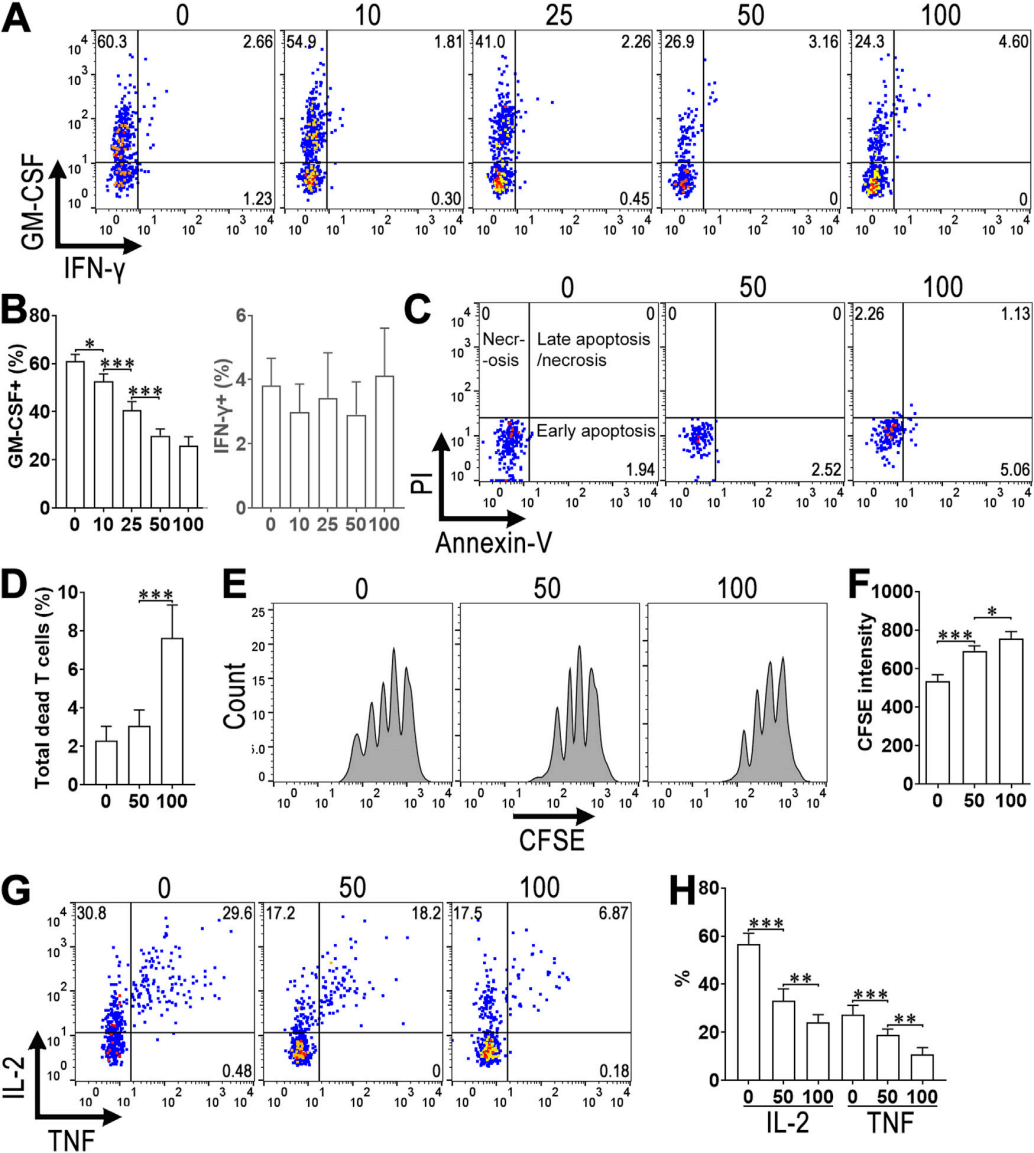
The effect of calycosin on *in vitro* ThGM cell differentiation. **(A)** Dot plots indicating the expression of GM-CSF and IFN-γ in splenic CD4^+^ T cells after *in vitro* ThGM differentiation. 0–100: 0–100 μM Calycosin. **(B)** The frequencies of GM-CSF^+^ (Left panel) and IFN-γ^+^ (Right panel) cells in differentiated ThGM cells. **(C)** Dot plots showing the apoptosis and necrosis of differentiated ThGM cells. **(D)** The frequency of total dead T cells (including early apoptotic, late apoptotic, and necrotic cells). **(E)** Histograms showing CFSE dilution in differentiated ThGM cells. **(F)** CFSE intensities in differentiated ThGM cells. **(G)** Dot plots indicating the expression of IL-2 and TNF in differentiated ThGM cells. **(H)** The frequencies of IL-2^+^ and TNF^+^ cells in differentiated ThGM cells. N = 6 samples per group. One-Way ANOVA. *: *P* < 0.05. **: *P* < 0.01. ***: *P* < 0.001.

The low cellularity of aortic ThGM cells limited further investigation of calycosin-induced effect on ThGM cells. Hence, we used resting splenic CD4^+^ T cells to induce ThGM cell generation *in vitro*. As demonstrated in Figures 4A,B, profound GM-CSF expression and few IFN-γ expression were found in CD4^+^ T cells, indicating ThGM cell differentiation. Calycosin reduced GM-CSF expression in a dose-dependent manner, with the maximum effect at 50 and 100 μM. Furthermore, 50 μM calycosin did not trigger T cell death while 100 μM calycosin caused mild apoptosis and necrosis of T cells (Figures 4C,D). Calycosin also significantly slowed ThGM cell proliferation, as evidenced by higher CFSE intensities of calycosin-treated ThGM cells (Figures 4E,F). Moreover, IL-2 and TNF, which are another two ThGM-associated cytokines, were downregulated by calycosin (Figures 4G,H). Therefore, calycosin impeded ThGM cell generation. We used 50 μM calycosin in the following experiments.

### 3.5 Calycosin impairs the macrophage-activating effect of ThGM cells *in vitro*

**FIGURE 5:**
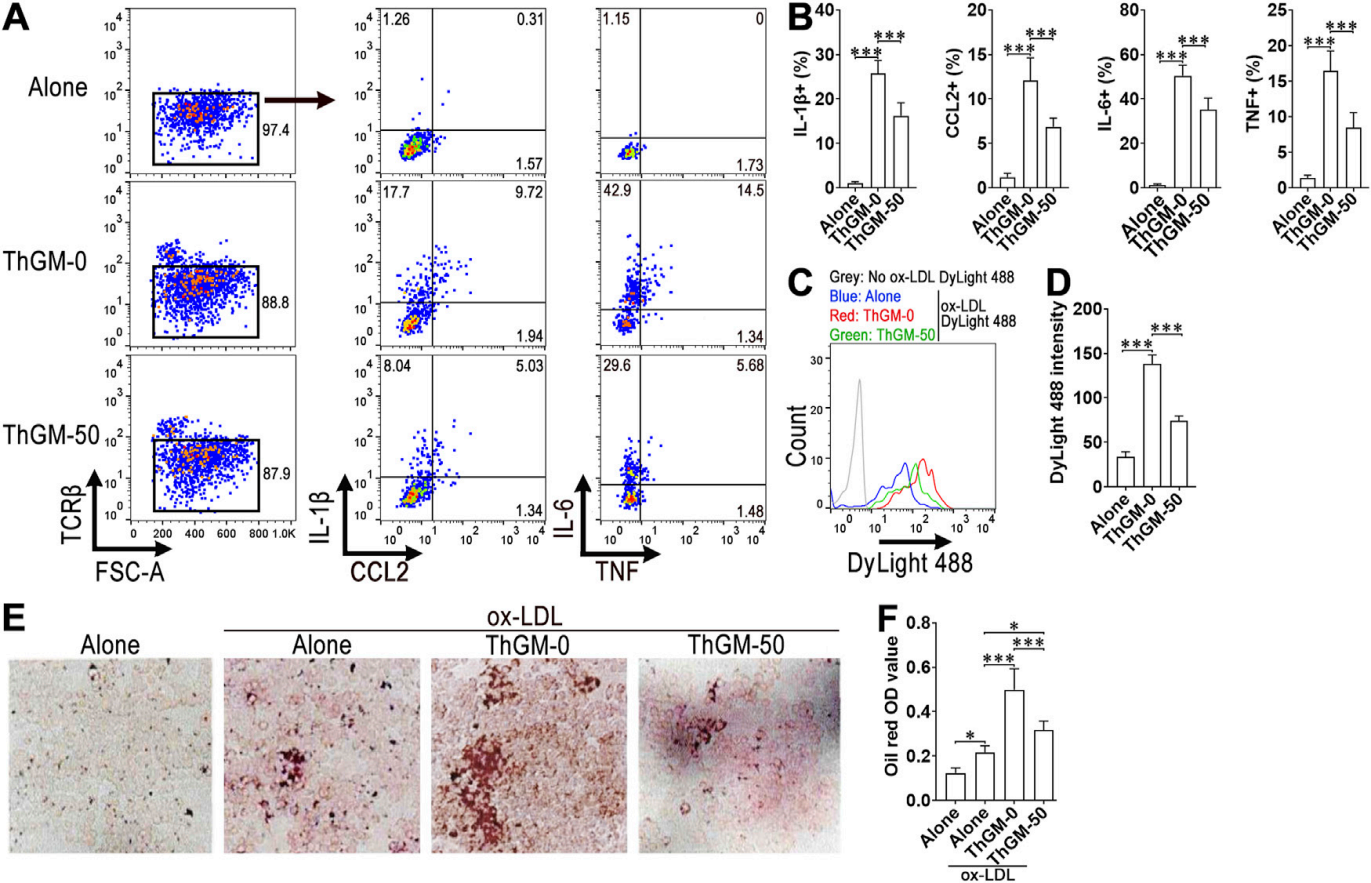
The influence of calycosin on ThGM cell-induced activation of macrophages. **(A)** Dot plots demonstrating the staining of IL-1β, CCL2, IL-6, and TNF in macrophages after co-culture with ThGM cells. Alone: macrophages alone. ThGM-0: macrophages cultured with vehicle-treated ThGM cells. ThGM-50: macrophages cultured with ThGM cells that were pre-treated with 50 μM calycosin. **(B)** The frequencies of IL-1β^+^, CCL2^+^, IL-6^+^, and TNF^+^ macrophages. **(C)** Histograms exhibiting intracellular oxLDL-DyLight 488 in macrophages after they were cultured with ThGM cells and then incubated with oxLDL-DyLight 488. No ox-LDL DyLight 488: without ox-LDL DyLight 488 incubation. **(D)** Intracellular DyLight 488 intensity in macrophages. **(E)** Oil red O staining of macrophages after 24-h co-culture with ThGM cells and then incubation with oxLDL. **(F)** Oil red O OD values. N = 6 samples per group. One-Way ANOVA. *: *P* < 0.05. ***: *P* < 0.001.

Vehicle- or calycosin-treated ThGM cells were cultured with resting macrophages for 24 h, followed by collecting macrophages to quantify intracellular IL-1β, CCL2, IL-6, and TNF. As displayed in Figures 5A,B, vehicle-treated ThGM cells substantially promoted the expression of these cytokines in macrophages, whereas calycosin-treated ThGM cells induced much lower cytokine production in macrophages. To determine the oxLDL uptake capacity of macrophages, macrophages were co-cultured with ThGM cells and then incubated with oxLDL DyLight 488. Compared with macrophages cultured alone, macrophages co-cultured with vehicle-treated ThGM cells engulfed more ox-LDL DyLight 488, indicating that ThGM cells enhanced the ox-LDL uptake capacity of macrophages (Figures 5C,D). However, after culture with calycosin-treated ThGM cells, macrophages exhibited a lower DyLight 488 intensity, suggesting that calycosin-treated ThGM cells could not promote macrophage ox-LDL uptake capacity as potently as vehicle-treated ThGM cells (Figures 5C,D). Furthermore, compared with macrophages cultured alone, macrophages cultured with vehicle-treated ThGM cells exhibited a higher oil red O staining value after ox-LDL exposure, implying that ThGM cells increased lipid content in macrophages. However, calycosin-treated ThGM cells were less potent in doing so (Figures 5E,F). Quantification of cholesterol content substantiated this result (Supplementary Figure S3). To determine whether exogenous GM-CSF can compensate for the weakened effect of ThGM cells on macrophages, we added GM-CSF (20 ng/mL) at the start of ThGM cell-macrophage co-culture. In the presence of GM-CSF, macrophages that were co-cultured with calycosin-treated ThGM cells expressed even higher IL-1β, CCL2, IL-6, and TNF than macrophages co-cultured with vehicle-treated ThGM cells (Supplementary Figures S4A,B). Similarly, in the presence of GM-CSF, macrophages that were co-cultured with calycosin-treated ThGM cells engulfed even more ox-LDL DyLight 488 than macrophages co-cultured with vehicle-treated ThGM cells (Supplementary Figures S4C,D). Therefore, calycosin impaired ThGM cell-mediated enhancement of macrophage pro-atherosclerotic function via decreasing GM-CSF production.

### 3.6 Calycosin increases NR4A3 expression to suppress ThGM cell function

**FIGURE 6:**
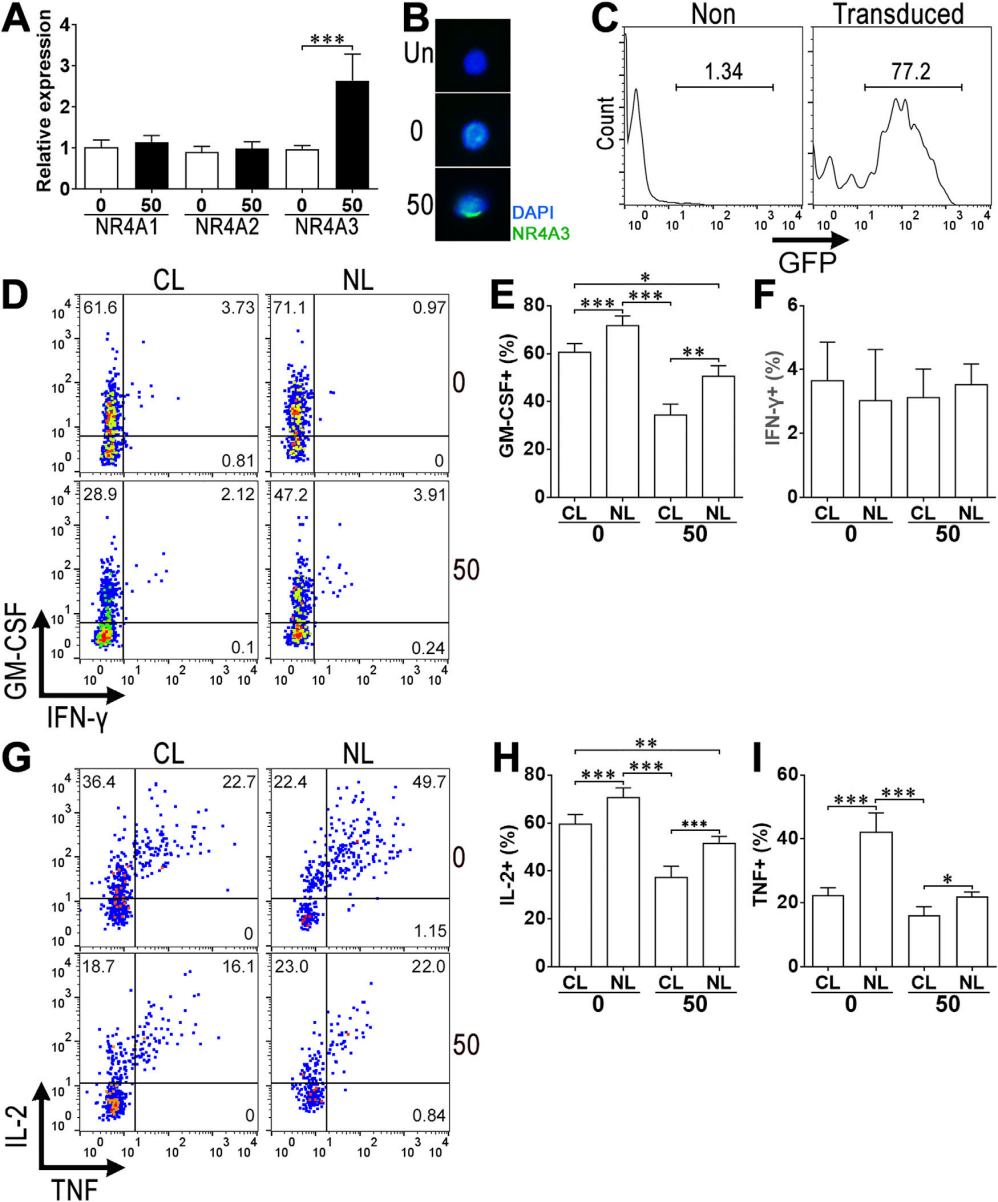
The role of NR4A3 in calycosin-induced changes. (A) mRNA levels of NR4A1, NR4A2, and NR4A3 in differentiated ThGM cells. 0: ThGM cell differentiation in the absence of calycosin. 50: ThGM cell differentiation in the presence of 50 μM calycosin. **(B)** Immunofluorescence microscopy of NR4A3 in differentiated ThGM cells. Un: Unstained (first antibody omitted) cells. **(C)** Representative histograms showing GFP expression in non-transduced ThGM cells (Non) and lentivirus-transduced ThGM cells (Transduced). **(D)** Dot plots presenting intracellular GM-CSF and IFN-γ in GFP^+^ ThGM cells CL: ThGM cells transduced with control lentivirus. NL: ThGM cells transduced with NR4A3-shRNA lentivirus. 0: No calycosin. 50: 50 μM calycosin. **(E,F)** Frequencies of GM-CSF^+^ **(E)** and IFN-γ^+^ **(F)** cells in GFP^+^ ThGM cells. **(G)** Dot plots representing the expression of IL-2 and TNF in GFP^+^ ThGM cells. **(H,I)** Frequencies of IL-2^+^ **(H)** and TNF^+^ **(I)** cells in GFP^+^ ThGM cells. N = 4 or 6 samples per group. Student’s t-test for **(A)**. One-Way ANOVA for **(E,I)**. *: *P* < 0.05. **: *P* < 0.01. ***: *P* < 0.001.

Calycosin upregulated the mRNA of NR4A3 but not NR4A1 and NR4A2 in ThGM cells in comparison with vehicle-treated ThGM cells (Figure 6A). Immunofluorescence microscopy and Immunoblotting confirmed higher NR4A3 expression in calycosin-treated ThGM cells (Figure 6B; Supplementary Figure S5). We silenced NR4A3 by transducing NR4A3-shRNA lentivirus into differentiating ThGM cells in the presence or absence of calycosin. The transduction efficiency was nearly 80% according to the proportion of GFP^+^ cells (Figure 6C), causing substantial downregulation of NR4A3 while not changing NR4A1 and NR4A2 expression (Supplementary Figure S6). The expression of ThGM-associated cytokines was then appraised in GFP^+^ ThGM cells (i.e., lentivirus-transduced cells). In the absence of calycosin, NR4A3 silencing increased GM-CSF expression compared with control virus-transduced cells. In calycosin-treated ThGM cells, NR4A3 silencing strongly restored GM-CSF expression (Figures 6D–F). Similarly, NR4A3 silencing also significantly increased IL-2 and TNF expression in ThGM cells in the absence or presence of calycosin (Figures 6G–I). Therefore, NR4A3 is an intrinsic suppressor of ThGM cells and calycosin affects ThGM cell function via up-regulating NR4A3.

## 4 Discussion

ThGM cells contribute to the physiopathological alterations of autoimmune neuroinflammation, endometriosis, adenomyosis, and autoimmune uveitis (Noster et al., 2014; Sheng et al., 2014; Lin et al., 2023; Wu et al., 2023; Zhang et al., 2024). In our previous study, we characterized the presence of aortic CD4^+^IFN-γ^-^GM-CSF^+^ T cells in atherosclerotic mice. Because these T cells are almost devoid of T-bet, GATA-3, RORγt, and Foxp3, they were identified as ThGM cells. In the present study, ThGM cells were also found in atherosclerotic aortas. It has been revealed that T cells are primed to exhibit activation phenotype in the aortic vessel wall (MacRitchie et al., 2020). Besides, IL-7, which is key to ThGM differentiation, exists in atherosclerotic lesions (Li et al., 2012). Multiple cell types including epithelial cells, stromal cells, and IL-1β-stimulated endothelial cells can secrete IL-7 (Bhaskar et al., 2011; Chen et al., 2021). Accordingly, perhaps IL-7 and unidentified antigen-presenting cells work together to induce CD4^+^ T cells to differentiate into ThGM cells in inflammatory atherosclerotic lesions.

Interestingly, calycosin attenuated the frequencies of aortic ThGM cells and Th1 cells, raising the possibility that calycosin suppresses T-cell-mediated chronic inflammation in atherosclerosis. Furthermore, IFN-γ expression was mildly decreased in aortic Th1 cells while GM-CSF expression was considerably alleviated in aortic ThGM cells, strongly suggesting that ThGM cells could be the primary target of calycosin relative to Th1 cells. Importantly, although aortic Th1 cell proliferation and function seemed to be mildly suppressed by calycosin, their activating effect on macrophages was only slightly (insignificantly) decreased, suggesting that calycosin-induced suppression of Th1 cell function was too weak to remarkably impair Th1 cell-mediated macrophage activation. By contrast, calycosin treatment elicited strong downregulation of ThGM cell proliferation and function, and subsequently profoundly impeded ThGM cell-induced macrophage activation. To our knowledge, we are the first to report the differential effects of calycosin on distinct CD4^+^ T cell subsets.

In our *in vitro* ThGM induction assay, the effects of calycosin on ThGm cells were similar to the *in vivo* changes, including lower ThGM-associated cytokine expression, slower proliferation, and weaker macrophage activation. Consistent with our previous research (Xiong et al., 2024), ThGM cells promoted the ox-LDL uptake capacity of macrophages and potently enhanced foam cell formation. By contrast, calycosin markedly lessened ThGM cell-mediated enhancement of ox-LDL uptake capacity of macrophages and thus mitigated foam cell formation as well as the cholesterol content. Therefore, calycosin likely not only directly acts on pro-atherosclerotic macrophages as formerly reported (Ma et al., 2022) but also suppresses macrophage-activating ThGM cells in the aorta.

Calycosin is a natural phytoestrogen and the principal effective factor of A. membranaceus. It has great potential in treating several disorders owing to its low toxicity and impressive efficiency. Therefore, it has attracted the attention and interest of pharmacologists and clinical researchers. Laboratory studies have identified anti-inflammatory, antioxidant, anti-tumor, and immune modulatory effects as the fundamental properties of calycosin’s efficacy. A number of signal pathways are responsible for the effects of calycosin, including but not limited to the Nrf2/SLC7A11/GPX4 signaling, AMPK/mTOR signaling, KLF2-MLKL-mediated autophagy pathway, MAPK, STAT3, and NF-κB signaling (Liu et al., 2021; Ma et al., 2022; Zhou et al., 2023; Han et al., 2024). In our study, we identified NR4A3 as a downstream target of calycosin. NR4A3 is a member of the NR4A subfamily. NR4A proteins act as transcription regulators and their activities are primarily modulated via their expression levels (Odagiu et al., 2020b). Importantly, NR4A proteins contribute to T cell anergy or exhaustion since they might induce T cell exhaustion (Yoshimura et al., 2021). Interestingly, we found that NR4A3 was regulated by calycosin, raising the question of how calycosin targets NR4A3. One possibility is that calycosin could directly bind to signaling molecules to boost NR4A3 expression. Recent molecular docking analysis suggests that calycosin interacts with mitogen-activated protein kinase-1 (MAPK1), mitogen-activated protein kinase 3 (MAPK3), IL-6, β-arrestin 1 (ARRB1), and homologue-1 (ABL1) (Xu et al., 2022; Xu et al., 2024). Interestingly, NR4A proteins can be regulated by MAPK pathways (Mohan et al., 2012). It is therefore possible that calycosin binds to and activates MAPKs to trigger NR4A3 upregulation. Furthermore, the expression of NR4A proteins is also influenced by protein kinase A/CREB, NF-κB, phosphoinositide 3-kinase/AKT, c-*jun*-NH_2_-kinase, and Wnt (Mohan et al., 2012; Bending and Zikherman, 2023). Calycosin would modulate these signaling cascades to increase NR4A3 expression. Our next goal is to evaluate the roles of these signal pathways in the expression of NR4A proteins. Besides, because calycosin is a potent autophagy inducer (Ma et al., 2022; Zhou et al., 2023), the impact of autophagy on ThGM cell activity should also be investigated in the future. The importance of NR4A3 for calycosin-induced effect also raises the possibility that the different sensitivities of ThGM cells and Th1 cells to calycosin might result from differential NR4A3 expression levels. Perhaps atherosclerotic ThGM cells express higher NR4A3 than atherosclerotic Th1 cells after calycosin exposure, making ThGM cells prone to anergy or exhaustion. If the differential NR4A3 expression does exist, it could be due to differential intensities of TCR signaling or the above-mentioned signaling pathways between ThGM cells and Th1 cells. However, this hypothesis has to be validated in the future.

According to former studies, there are significant sex differences in the pathology of atherosclerosis (Poznyak et al., 2023). Estrogen may protect the cardiovascular system through promoting vasodilation, reducing LDL cholesterol, and maintaining endothelial function (Iorga et al., 2017). Besides, although there is no hard evidence demonstrating the effect of estrogen on ThGM cells, estrogen has been shown to positively or negatively modulate the differentiation and functions of Th1 and Th17 cells depending on the context (Fuseini et al., 2019; Alanazi et al., 2024), suggesting the potential impact of estrogen on ThGM cells. Furthermore, estrogen levels fluctuate during the menstrual cycle (Farage et al., 2009), possibly causing non-negligible variations in atherosclerosis development or ThGM cell function in female mice. The levels of testosterone, however, are relatively stable in age-matched inbred male mice and might contribute to atherosclerosis progression (Gencer et al., 2021). Therefore, we used male mice only in this study to avoid potential estrogen-induced variations in either atherosclerosis development or ThGM cell function. The impact of calycosin on ThGM cells of female mice, however, deserves further investigation.

## 5 Conclusion

In summary, this study unveils the modulatory effects of calycosin on aortic ThGM cell function and subsequent macrophage activation in atherosclerosis, highlighting a novel immunoregulatory and protective mechanism of calycosin in atherosclerosis treatment. It would be beneficial to corroborate our findings in human specimens to deepen the understanding of the prospective pharmacological properties of calycosin.

## Data Availability

The raw data supporting the conclusions of this article will be made available by the authors, without undue reservation.

## Abbreviations

ApoE, apolipoprotein E; CCL, C-C motif chemokine ligand; CCR, C-C chemokine receptor; CXCR, C-X-C Motif Chemokine Receptor; GM-CSF, granulocyte-macrophage-colony-stimulating factor; IFN-γ, interferon-gamma; IL, interleukin; ox-LDL, oxidized low-density lipoprotein; NR4A, nuclear receptor subfamily 4 group A; RORγt, retinoic acid-related orphan receptor gamma t; T-bet, T-box expressed in T cells; Th1 cells, T helper 1 cells; ThGM cells. GM-CSF-producing T helper cells, TNF, tumor necrosis factor.
