# Supplementary material for: Calycosin suppresses the activating effect of granulocyte-macrophage-colony-stimulating factor-producing T helper cells on macrophages in experimental atherosclerosis
Source: Front Pharmacol. 2025 Jul 10;16:1607349. doi: 10.3389/fphar.2025.1607349 (PMC12286824; doi:10.3389/fphar.2025.1607349)
Supplement: Supplementary file 1 [file Supplementaryfile1.docx]

| **Supplementary Table 1. Flow cytometry antibodies** | | |
| --- | --- | --- |
| **Name** | **Clone #** | **Vendor** |
| Pacific blue anti-TCRβ | H57-597 | BioLegend |
| PE anti-CD4 | GK1.5 | BioLegend |
| APC anti-IFN-γ | XMG1.2 | BioLegend |
| FITC anti-GM-CSF | MP1-22E9 | BioLegend |
| PE/Cy7 anti-GM-CSF | MP1-22E9 | BioLegend |
| PerCP/Cyanine5.5 anti-CXCR3 | CXCR3-173 | BioLegend |
| Brilliant Violet 711 anti-CCR8 | SA214G2 | BioLegend |
| PE/Cy7 anti-CCR6 | 29-2L17 | BioLegend |
| FITC anti-Ki67 | 11F6 | BioLegend |
| APC anti-IL-2 | JES6-5H4 | BioLegend |
| Alexa Fluor 750 anti-CCR10 | 248918 | R&D |
| PE anti-IL-1β | 166931) | R&D |
| PE anti-TNF | MP6-XT22 | ThermoFisher |
| FITC anti-CCL2 | 2H5 | ThermoFisher |
| FITC anti-IL-6 | MP5-20F3 | eBioscience |

| **Supplementary Table 2. Primers** | | |
| --- | --- | --- |
| **Gene** | **Sense 5’ to 3’** | **Anti-sense 5’ to 3’** |
| *Actb* | AGAGGGAAATCGTGCGTGAC | CAATAGTGATGACCTGGCCGT |
| *Il1b* | TGGACCTTCCAGGATGAGGACA | GTTCATCTCGGAGCCTGTAGTG |
| *Il6* | TACCACTTCACAAGTCGGAGGC | CTGCAAGTGCATCATCGTTGTTC |
| *Tnf* | GGTGCCTATGTCTCAGCCTCTT | GCCATAGAACTGATGAGAGGGAG |
| *Ccl2* | GCTACAAGAGGATCACCAGCAG | GTCTGGACCCATTCCTTCTTGG |
| *Nr4a1* | GTGCAGTCTGTGGTGACAATGC | CAGGCAGATGTACTTGGCGCTT |
| *Nr4a2* | CCGCCGAAATCGTTGTCAGTAC | TTCGGCTTCGAGGGTAAACGAC |
| *Nr4a3* | ACGCCGAAACCGATGTCAGTAC | CTCCTGTTGTAGTGGGCTCTTTG |


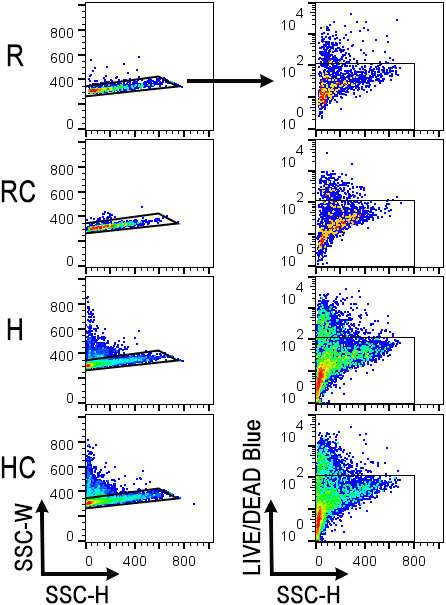


**Supplementary Figure 1. Dot plots indicating the gating strategy for single cells and live cells among recovered aortic leukocytes.** R: ApoE^-/-^ mice fed a regular diet. RC: ApoE^-/-^ mice fed the calycosin-containing regular diet. H: ApoE^-/-^ mice fed the high-fat diet. HC: ApoE^-/-^ mice fed the calycosin-containing high-fat diet.

**
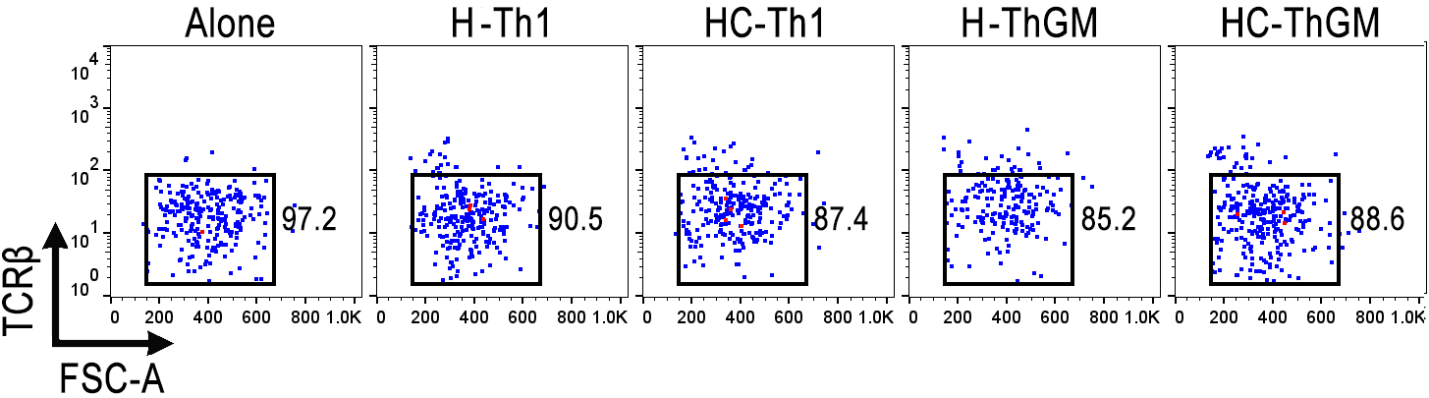
**

**Supplementary Figure 2. Dot plots demonstrating how to sort macrophages after co-culture with ThGM cells followed by removing floating ThGM cells.** Alone: macrophages alone. H-Th1: macrophages co-cultured with Th1 cells of mice fed the high-fat diet. HC-Th1: macrophages co-cultured with Th1 cells of mice fed the calycosin-containing high-fat diet. H-ThGM: macrophages co-cultured with ThGM cells of mice fed the high-fat diet. HC-ThGM: macrophages co-cultured with ThGM cells of mice fed the calycosin-containing high-fat diet.


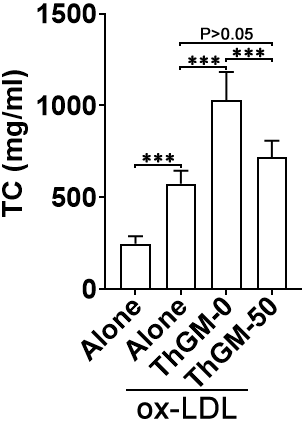


**Supplementary Figure 3. Total cholesterol in macrophages.** Alone: macrophages alone. ThGM-0: macrophages cultured with vehicle-treated ThGM cells. ThGM-50: macrophages cultured with ThGM cells that were pre-treated with 50 μM calycosin. N=6 samples per group. One-Way ANOVA. ***: *P*<0.001.


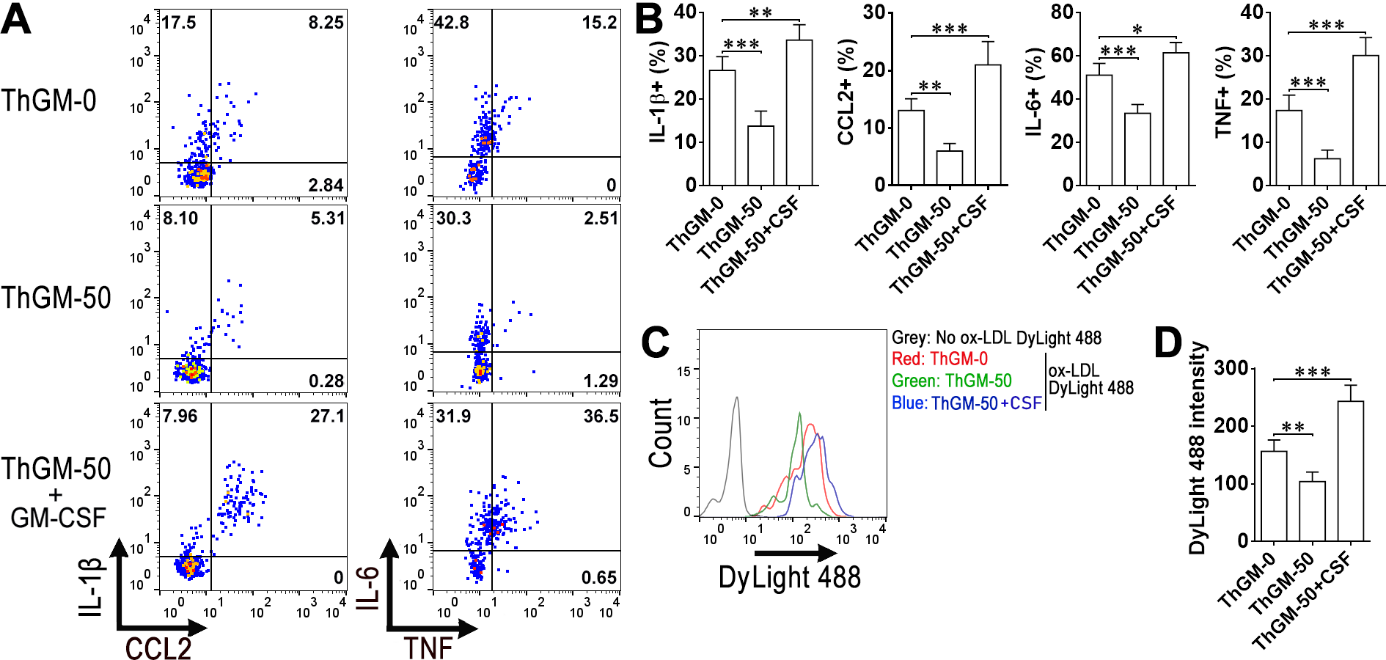


**Supplementary Figure 4. GM-CSF boosts the activating effect of calycosin-treated ThGM cells on macrophages. (A)** Dot plots demonstrating the staining of IL-1β, CCL2, IL-6, and TNF in macrophages after co-culture with ThGM cells. ThGM-0: macrophages cultured with vehicle-treated ThGM cells. ThGM-50: macrophages cultured with ThGM cells that were pre-treated with 50 μM calycosin. ThGM-50+CSF: macrophages cultured with calycosin-pre-treated ThGM cells in the presence of recombinant GM-CSF. **(B)** The frequencies of IL-1β^+^, CCL2^+^, IL-6^+^, and TNF^+^ macrophages. **(C)** Histograms exhibiting intracellular oxLDL-DyLight 488 in macrophages after they were cultured with ThGM cells and then incubated with oxLDL-DyLight 488. No ox-LDL DyLight 488: without ox-LDL DyLight 488 incubation. **(D)** Intracellular DyLight 488 intensity in macrophages. N=5 or 6 samples per group. One-Way ANOVA. *: *P*<0.05. **: *P*<0.01. ***: *P*<0.001.


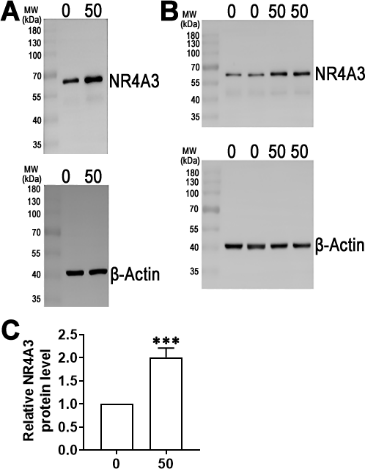


**Supplementary Figure 5. NR4A3 protein expression in differentiated ThGM cells after calycosin treatment. (A and B)** Representative Immunoblotting images. **(C)** Relative NR4A3 protein levels. 0: ThGM cell differentiation in absence of calycosin. 50: ThGM cell differentiation in the presence of 50 μM calycosin. N=3 samples per group. Student’s t-test. ***: *P*<0.001.

**
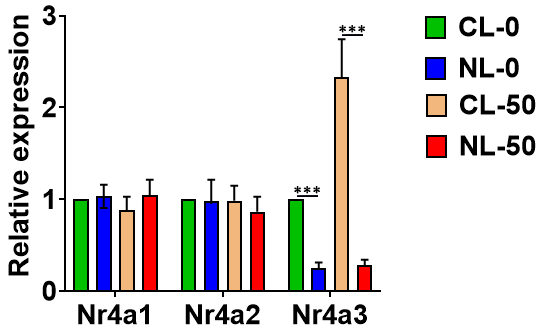
**

**Supplementary Figure 6. mRNA levels of NR4A1, NR4A2, and NR4A3 in ThGM cells after lentiviral transduction.** CL: ThGM cells transduced with control lentivirus. NL: ThGM cells transduced with NR4A3-shRNA lentivirus. 0: No calycosin. 50: 50 μM calycosin. N=3 samples per group. ***: *P*<0.001. Student’s t-test.
